# Upper Limb Function in People With Upper and Lower Limb Loss 8 Years Postinjury: The Armed Services Trauma Outcome Study (ADVANCE) Cohort Study

**DOI:** 10.1093/ptj/pzae082

**Published:** 2024-07-02

**Authors:** Fraje C E Watson, Angela E Kedgley, Susie Schofield, Fearghal P Behan, Christopher J Boos, Nicola T Fear, Alexander N Bennett, Anthony M J Bull

**Affiliations:** Department of Bioengineering, Imperial College London, London, UK; Department of Bioengineering, Imperial College London, London, UK; National Heart and Lung Institute, Faculty of Medicine, Imperial College London, London, UK; Department of Bioengineering, Imperial College London, London, UK; Discipline of Physiotherapy, School of Medicine, Trinity College Dublin, Dublin, Ireland; Faculty of Health and Social Sciences, Bournemouth University, Poole, UK; King’s Centre for Military Research, King’s College London, London, UK; Department of Bioengineering, Imperial College London, London, UK; Academic Department of Military Rehabilitation, Defence Military Rehabilitation Centre, Loughborough, UK; Department of Bioengineering, Imperial College London, London, UK

**Keywords:** Amputation, Anatomy: Lower Extremity, Anatomy: Upper Extremity: Arm, Anatomy: Upper Extremity: Hand, Anatomy: Upper Extremity: Shoulder, Blast Injuries, Military Personnel, Musculoskeletal Diseases

## Abstract

**Objective:**

Upper limb (UL) disability in people with UL loss is well reported in the literature, less so for people with lower limb loss. This study aimed to compare UL disability in injured (major trauma) and uninjured UK military personnel, with particular focus on people with upper and lower limb loss.

**Methods:**

A volunteer sample of injured (*n* = 579) and uninjured (*n* = 566) UK military personnel who served in a combat role in the Afghanistan war were frequency matched on age, sex, service, rank, regiment, role, and deployment period and recruited to the Armed Services Trauma Rehabilitation Outcome (ADVANCE) longitudinal cohort study. Participants completed the Disability of the Arm, Shoulder, and Hand (DASH) questionnaire, scored from 0 (no disability) to 100 (maximum disability) 8 years postinjury. Mann–Whitney U and Kruskal–Wallis tests were used to compared DASH scores between groups. An ordinal model was used to assess the effect of injury and amputation on DASH scores.

**Results:**

DASH scores were higher in the Injured group compared to the Uninjured group (3.33 vs 0.00) and higher in people with lower limb loss compared to the Uninjured group (0.83 vs 0.00), although this was not statistically significant. In the adjusted ordinal model, the odds of having a higher DASH score was 1.70 (95% CI = 1.18–2.47) times higher for people with lower limb loss compared to the Uninjured group. DASH score was not significantly different between people with major and partial UL loss (15.42 vs 12.92). The odds of having a higher DASH score was 8.30 (95% CI = 5.07–13.60) times higher for people with UL loss compared to the Uninjured group.

**Conclusion:**

People with lower limb loss have increased odds of having more UL disability than the Uninjured population 8 years postinjury. People with major and partial UL loss have similar UL disability. The ADVANCE study will continue to follow this population for the next 20 years.

**Impact:**

For the first time, potential for greater long-term UL disability has been shown in people with lower limb loss, likely resulting from daily biomechanical compensations such as weight-bearing, balance, and power generation. This population may benefit from prophylactic upper limb rehabilitation, strength, and technique.

## Introduction

The upper limbs (ULs) are integral to performing activities of daily living and provide a means for communication and self-expression.^1^^,^^2^ High levels of UL disability in people with major (proximal to the wrist including wrist disarticulation) and partial (distal to the wrist) UL loss have been reported in military^3^^,^^4^ and civilian populations.^5–7^ Disability in people with partial UL loss is extremely varied depending on the level of amputation and thumb involvement.^4^^,^^5^^,^^8–10^ Most research on people with partial UL loss focuses on outcomes compared to replantation^8–10^ and not compared to people with major UL loss who may have better access to rehabilitation and prosthetic devices. Following initial injury, people with major UL loss have increased odds of an UL overuse musculoskeletal injury in the first year postamputation^11^ and chronic injury to the contralateral limb,^12^ which could further compound initial disability. Despite the lifelong impact of UL amputation coupled with the consequences of biomechanical compensations and aging, a prospective cohort has never been followed longitudinally beyond medical discharge from hospital care.

People with lower limb loss have a 2 to 4 times increased risk of UL musculoskeletal injury 1 year postinjury compared to those with minor lower limb injuries.^13^ These short-term findings mirror long-term UL musculoskeletal injuries reported in wheelchair and assistive walking device users, resulting from increased forces and altered biomechanics through the UL joints during propulsion/ambulation and transfer activities.^14–16^ Little is known about the long-term progression and impact of increased UL musculoskeletal injuries on people with lower limb loss.

The Armed Services Trauma Rehabilitation Outcome Study (ADVANCE) is a 20 year cohort study collecting physical and psychosocial outcome data from 1145 male UK military personnel who deployed to the Afghanistan war between 2003 and 2014.^17^ Approximately half of the cohort were severely physically injured requiring aeromedical evacuation to a UK hospital. The most common mechanism of injury is blast, so many of this cohort experienced multiple serious injuries including traumatic amputation. Uninjured personnel were frequency matched on age, service, rank, regiment, role, and deployment period. Data will be collected at 6 timepoints over 20 years.

The aims of this study were to test the following hypotheses in the Injured group is greater than the Uninjured (control) group; (1) UL disability in people with lower limb loss is greater than the Uninjured group; (2) UL disability in people with major UL loss is greater than in people with partial UL loss; and (3) UL disability in people with major and partial UL loss is greater than the Uninjured group and remaining Injured subgroups.

## Methods

### Recruitment & Study Participants

Participants were recruited from Defence Statistics UK lists.^17^ The Injured and Uninjured cohort were males aged >18 and <50 years. Exclusion criteria were a diagnosis of cardiac disease, diabetes, renal disease, or liver disease prior to injury or deployment of interest to ensure long-term outcomes could be attributed to combat injury instead of potential preexisting conditions. The Uninjured group did not sustain subsequent combat injury requiring aeromedical evacuation before or after matching. There were very few female UK military combat casualties such that sufficiently powered or translatable results could not be drawn.

Ethical approval was granted by the Ministry of Defence Research Ethics Committee in January 2013 (protocol no: 357/PPE/12).

### Procedure

Participants gave informed consent and attended data collection at the Defence Medical Rehabilitation Centre Headley Court (March 2016–August 2018) or Stanford Hall (August 2018 onward) for comprehensive health tests and questionnaires.^17^

### Questionnaire Assessment

The Disability of the Arm, Shoulder, and Hand (DASH) questionnaire is an assessment of UL disability^18^^,^^19^ consisting of a Disability/Symptom module followed by optional Work and Sport/Music modules, which will not be described here. Responders rate their ability to perform 21 daily activities (eg, wash their hair, use a knife to cut food) in the last week on a scale from 1 (no difficulty) to 5 (unable), followed by 9 questions about the impact of any UL challenges.

The DASH questionnaire is valid when ≥27 questions have been answered and is calculated by dividing the sum of scores by the number of scores, subtracting 1 and multiplying by 25.^20^ The final scale is from 0 (no disability) to 100 (greatest disability). The minimum clinically important difference (MCID) is 10.8.^21^

Handedness was assessed retrospectively for people with major UL loss only. Participants answered 3 questions from the Edinburgh Handedness Inventory^22^ about handedness prior to their injury: which hand they used for (1) writing, (2) throwing, and (3) holding a knife to cut bread. Responses were “always right” (2 points right), “always left” (2 points left), “usually right” (1 point right), “usually left” (1 point left), or “both equally” (1 point right and left). Results were calculated by dividing scores for right minus left by the sum of right and left, then multiplying by 100 to categorize participants as purely right (≥60), mixed right (≤20 and <60), neutral (< 20 and ≤ −20), mixed left (< −20 and ≥ −60), and purely left (< −60).

### Study Variables

Participants were grouped as Injured or Uninjured, as described above. The Injured group was subdivided into Injured—Non-Amputee (Inj-NA), Injured—Major Lower Limb Loss (Inj-LL), Injured—Major UL Loss (Inj-ULmajor), and Injured—Partial UL Loss (Inj-ULpartial). Participants with upper and lower limb loss in combination were grouped as Inj-ULmajor or Inj-ULpartial so that concurrent UL amputations did not affect conclusions about UL disability in people with lower limb loss.

The Abbreviated Injury Score (AIS) gives a score of 1 (minor) to 6 (maximal) for the extent of injury at a single body location.^23^ The New Injury Severity Score (NISS) is the sum of the squares of the 3 highest AISs regardless of body region and has a maximum score of 75.^24^ Socioeconomic status was classified using military rank at the time of deployment equating to a 3-tier National Statistics Socioeconomic Classification (NS-SEC); senior ranks are group 1 (eg, Commissioned Officer), mid-ranks are group 2 (eg, Senior Non-Commissioned Officer), and junior ranks are group 3 (eg, Junior Non-Commissioned Officer).^25^^,^^26^ Race was classified as White, Black, Asian, and Other.

### Statistical Analysis

Thirteen participants were excluded from the analysis, including 11 with invalid DASH scores (3 Uninjured, 8 Injured), 1 with a partial UL loss classified as a minor combat injury, and 1 with noncombat-related lower limb loss, both of whom met the criteria for the Uninjured group. Multiple imputation was not used because data loss was minimal and only in the outcome measure.

An *a priori* power analysis was conducted according to the study protocol.^17^ Normality of continuous variables were assessed by visual inspection. The Mann–Whitney U test was used to compare nonparametric continuous variables between 2 groups (eg, Injured group vs Uninjured group). The Kruskal–Wallis test with Bonferroni correction was used to compare nonparametric continuous variables between 3 or more groups with a prespecified subgroup analysis comparing Uninjured versus Inj-LL; Inj-ULmajor versus Inj-ULpartial, Inj-LL, Inj-NA and Uninjured; and Inj-ULpartial versus Inj-LL, Inj-NA, and Uninjured groups based on the aforementioned hypotheses. Additional post hoc comparisons using a Bonferroni correction were carried out to test the remaining relationships (Uninjured vs Inj-NA and Inj-NA vs Inj-LL). To model the association between exposure and DASH score, we fitted a cumulative probability model (CPM) with a logit link (proportional odds model). The DASH score is a nonparametric semicontinuous outcome, and the CPM is a flexible model that can be used for skewed continuous and semicontinuous outcomes.^27^ Age, race, and rank at sampling were included as *a priori* confounding variables and were controlled for in the model as they are known to affect DASH.^28–30^ To relax the strong assumption of linearity, age was modeled using restricted cubic splines with 4 knots. Odds ratios and their 95% CIs are reported and can be interpreted as the odds of having a higher score on DASH for the Injured group compared to the Uninjured group.^31^ For the subgroup model, the Inj-ULmajor and Inj-ULpartial groups were combined due to small numbers and called Inj-UL. Model fit for ordinal models is often assessed by visually inspecting the Q-Q plot of the probability scale residuals (PSRs); however, since the outcome DASH is a mixture of discrete and continuous distributions, the Q-Q plot is not useful to assess the model fit due to the nonuniformly distributed PSRs. Alternatively, using PSRs in residual-by-predictor plots can detect lack of fit and were visually inspected^25^; plots were similar for probit and logit links, and the loglog link showed poorer fit. Therefore a, logit link was used.^27^ Statistical tests were undertaken with an alpha level of 0.05, taking into account Bonferroni correction where post hoc tests were performed. Statistical analysis was carried out in Stata version 17 (StataCorp LLC; College Station, Texas, USA) and using the add-on packages PResiduals and rms in R studio version 2023.03.1 (RStudio; Boston, MA, USA).

### Role of the Funding Source

The funder played no role in the design, conduct, or reporting of this study.

## Results

### Participant Demographics

Of the 1132 included participants, 571 (50.4%) were Injured. Participants were aged 34.1 (5.4) years at assessment, and the Injured group was 8.3 (2.2) years postinjury. Mean height and weight were 178.9 (6.4) cm and 87.9 (12.3) kg for the Uninjured group and 179.4 (7.1) cm and 90.5 (14.2) kg for the Injured group with adjusted weight values for people with limb loss. Blast injury accounted for 69.2% of injuries overall, but more than 93% of injuries in people with limb loss. Table 1 contains comprehensive demographic information.

**Table 1:** Participant Demographic Information for all Studied Groups*^a^*

**Variable**	**Uninjured** **(*n* = 561)**	**All Injured** **(*n* = 571)**	**Inj-NA** **(*n* = 404)**	**Inj-LL** **(*n* = 109)**	**Inj-ULmajor** **(*n* = 16)**	**Inj-ULpartial** **(*n* = 42)**
Age at sampling (y)	26.5 (5.3)	25.8 (5.2)	25.8 (5.4)	25.6 (4.8)	25.1 (4.8)	25.4 (5.1)
Age at assessment (y)	34.3 (5.4)	34.0 (5.4)	34.4 (5.5)	33.2 (4.7)	32.6 (4.3)	32.8 (5.4)
Time between injury and assessment (y)	–	8.3 (2.2)	8.6 (2.2)	7.6 (2.0)	7.5 (1.3)	7.4 (1.8)
Cause of injury Blast Gunshot Other	–	395 (69.2) 132 (24.9) 4 (0.8)	236 (58.4) 124 (34.1) 4 (1.1)	103 (94.5) 6 (5.5) 0 (0.0)	15 (93.8) 1 (6.3) 0 (0.0)	41 (97.6) 1 (2.4) 0 (0.0)
Height (cm)	178.9 (6.4)	179.4 (7.1)	179.0 (6.7)	180.1 (8.3)	180.4 (5.3)	180.9 (8.2)
Mass*^b^* (kg)	87.9 (12.3)	90.5 (14.2)	89.7 (13.8)	94.7 (14.6)	91.1 (12.8)	87.6 (15.5)
BMI*^b^* (kg/m^2^)	27.5 (3.4)	28.1 (3.9)	28.0 (3.7)	29.3 (4.2)	28.4 (4.2)	27.0 (4.5)
Race (White)	490 (87.3)	509 (89.1)	358 (88.6)	99 (90.8)	15 (93.8)	37 (88.1)
NISS (median, 25th–75th percentile)	–	12 (5–22)	9 (4–17)	22 (13–27)	34 (27–41)	29 (17–36)
NS-SEC Senior rank Mid-rank Junior rank	79 (14.1) 146 (26.0) 336 (59.9)	59 (10.3) 105 (18.4) 407 (71.3)	44 (10.9) 82 (20.3) 278 (68.8)	7 (6.4) 15 (13.8) 87 (79.8)	1 (6.3) 2 (12.5) 13 (81.3)	7 (16.7) 6 (14.3) 29 (69.1)
Still serving in military	463 (82.5)	154 (27.0)	137 (33.9)	8 (7.3)	1 (6.3)	8 (19.1)

^a^ Groups studied: Uninjured, all Injured, Inj-NA (Injured—Non-Amputee), Inj-LL (Injured—Major Lower Limb Loss), Inj-ULmajor (Injured—Major Upper Limb Loss), and Inj-ULpartial (Injured—Partial Upper Limb Loss). Data are presented as mean (SD) or number (%), unless otherwise stated. BMI = body mass index; NISS = New Injury Severity Score; NS-SEC = National Statistics—Socioeconomic Classification.

^b^ Adjusted for people with limb loss.

### Demographics of People With Major UL Loss

Inj-ULmajor participants had shoulder disarticulation (*n* = 1; 6.2%), transhumeral amputation (*n* = 4; 25.0%), and transradial amputation (*n* = 11; 68.8%). Amputation combinations are provided in Table 2.

**Table 2:** Details of Number of Participants With Isolated or Combination Upper and Lower Limb Loss in the Inj-LL, Inj-ULmajor, and Inj-ULpartial Groups*^a^*

**Group**	**No Lower Limb Loss**	**Unilateral Lower Limb Loss**	**Bilateral Lower Limb Loss**	**Total**
Inj-LL	–	70 (64.2%)	39 (35.8%)	109
Inj-ULmajor	3 (18.8%)	1 (6.3%)	12 (75.0%)	16
Inj-ULpartial	11 (26.2%)	6 (14.3%)	25 (59.5%)	42

^a^ Inj-LL = Injured—Major Lower Limb Loss; Inj-ULmajor = Injured—Major Upper Limb Loss; Inj-Ulpartial = Injured—Partial Upper Limb Loss.

Thirteen (81.3%) Inj-ULmajor participants reported using a UL prosthesis for activities of daily living (*n* = 8) and/or sport/exercise (*n* = 8). The participants who reported not using a UL prosthesis were people with bilateral lower limb and unilateral UL loss (*n* = 2) and a person with unilateral UL and ipsilateral unilateral lower limb loss (*n* = 1), all of whom used lower limb prostheses.

Handedness data were available for 13 (81.3%) participants in the Inj-ULmajor group, of whom 11 had reported using a prosthesis. Twelve were pure right-handers, and 1 was neutral. For the 11 prosthesis users, the dominant UL was amputated for 7 participants, the nondominant UL was amputated for 3 participants, and 1 participant was neutral.

UL injuries sustained by the Inj-NA and Inj-LL groups and their DASH scores are included in the Supplementary Table A.

### DASH Questionnaire

#### Uninjured and Injured Participants

DASH scores were higher in the Injured group compared to the Uninjured group (3.33 vs 0.00; *P* < .001) but did not meet the threshold for MCID (Figure).

**Figure:**
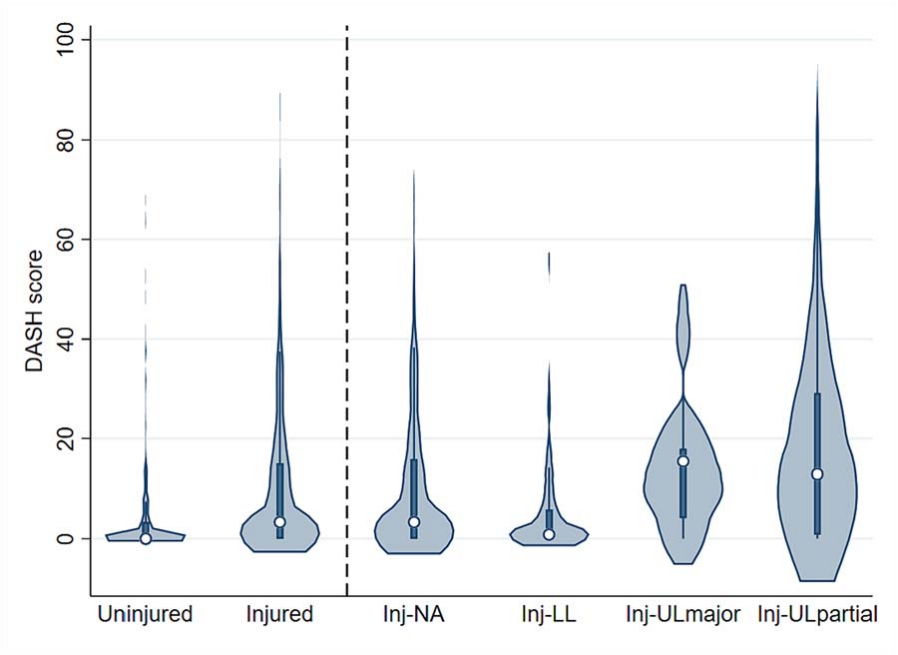
Violin plots for DASH scores for the Uninjured group and Injured group (left of the dashed line) and the Injured subgroups Inj-NA, Inj-LL, Inj-ULmajor, and Inj-ULpartial (right of the dashed line) 8 years postinjury (or matched deployment of interest). DASH = Disability of the Arm, Shoulder, and Hand questionnaire; Inj-LL = Injured—Lower Limb Loss; Inj-NA = Injured—Non-Amputee; Inj-ULmajor = Injured —Major Upper Limb Loss; Inj-ULpartial = Injured—Partial Upper Limb Loss.

#### Sub-Group Analysis

DASH scores were significantly different across subgroups (*P* < .001) (Figure, Tab. 3). Subgroup analyses showed strong evidence of a difference between the following subgroups: DASH was higher in both the Inj-ULmajor and Inj-ULpartial groups compared to the Uninjured (15.42 vs 0.00, *P* < .001 and 12.92 vs 0.00, *P* < .001, respectively) and Inj-LL groups (15.42 vs 0.83 *P* = .002 and 12.92 vs 0.83, *P* < .001, respectively). All differences met the threshold for MCID.

**Table 3:** DASH Scores for Uninjured, Inj-NA, Inj-LL, Inj-ULmajor and Inj-ULpartial Participants 8 Years Postinjury (or Matched Deployment of Interest)*^a^*

**DASH**	**Uninjured**	**Inj-NA**	**Inj-LL**	**Inj-ULmajor**	**Inj-ULpartial**
*n*	561	404	109	16	42
Median	0.00*^b,c,d^*	3.33*^b,e,f^*	0.83*^g,h^*	15.42*^c,e,g^*	12.92*^d,f,h^*
Range	0.00–68.33	0.00–70.00	0.00–55.83	0.00–44.17	0.00–86.67

^a^ DASH = Disability of the Arm, Shoulder, and Hand questionnaire; Inj-LL = Injured—Major Lower Limb Loss; Inj-NA = Injured—Non-Amputee; Inj-ULmajor = Injured—Major Upper Limb Loss; Inj-ULpartial = Injured—Partial Upper Limb loss.

^b-h^ Pairs of letters show where *P* < .05 for all preplanned and post hoc Injured group subanalysis with Bonferroni correction.

The small, nonsignificant difference in DASH scores between Inj-LL and the Uninjured group (0.83 vs 0.00; *P* = .06) did not meet the threshold for MCID, and there was no evidence of a difference between Inj-ULmajor and Inj-ULpartial (15.42 vs 12.92; *P* = 1.00).

Median DASH score for Inj-ULpartial participants with an amputation involving their thumb (*n* = 8) was 26.67 (range = 0.00–56.67) and 11.87 (range = 0.00–86.67) for those without an amputation involving their thumb (*n* = 34) (see Supp. Table B).

#### Regression Analysis

After adjustment for confounders, the odds of having a higher DASH score was 2.75 (95% CI = 2.20–3.43) times higher for participants that were Injured versus Uninjured participants (Tab. 4). In the subgroup analysis and after adjustment for confounders, compared to Uninjured participants, the odds of having a higher DASH score was 2.74 (95% CI = 2.15–3.50), 1.70 (95% CI = 1.18–2.47), and 8.30 (95% CI = 5.07–13.60) times higher for Inj-NA participants, Inj-LL participants, and Inj-UL participants, respectively (Tab. 4).

**Table 4:** Odds Ratio From Predictive Odds Ordinal Regression Analysis of DASH Scores for Overall Injury Status (Model 1) and Injury Status Subgroups (Model 2)*^a^*

**Predictor Variable**	**Unadjusted Odds Ratio (95% CI)**	**Adjusted Model 1** ^***b***^		**Adjusted Model 2** ^***c***^	
		**Odds Ratio (95% CI)**	***P***	**Odds Ratio (95% CI)**	***P***
Injury status Uninjured Injured	1 (ref) 2.72 (2.18–3.39)	1 (ref) 2.75 (2.20–3.43)	<.001	–	–
Injury status Uninjured Inj-NA Inj-LL Inj-UL	1 (ref) 2.75 (2.16–3.50) 1.65 (1.14–2.38) 8.03 (4.91–13.14)	–	–	1 (ref) 2.74 (2.15–3.50) 1.70 (1.18–2.47) 8.30 (5.07–13.60)	<.001

^a^ DASH = Disability of the Arm, Shoulder, and Hand questionnaire; Inj-LL = Injured—Major Lower Limb Loss; Inj-NA = Injured—Non-Amputee; Inj-UL = Injured—Upper Limb Loss; ref = reference.

^b^ Uninjured [*n* = 561], Injured [*n* = 571].

^c^ Uninjured [*n* = 561], Inj-NA [*n* = 404], Inj-LL [*n* = 109], and Inj-UL [*n* = 58].

## Discussion

As expected, people with major and partial UL loss had significantly more UL disability than Injured–Non-Amputees, participants with lower limb loss, and the Uninjured group. Participants with partial UL loss reported similar levels of UL disability to participants with major UL loss, suggesting UL disability is linked to full or partial loss of the hand (and possibly the thumb in particular) regardless of perceived injury severity. While the difference between participants with lower limb loss and the Uninjured group was very small and did not meet the MCID, adjusted analysis showed significantly increased odds (1.70) for a higher DASH score. The ADVANCE study provides a unique opportunity to monitor this cohort for the next 20 years.

Research describing UL disability in people with lower limb loss is sparse. A retrospective study of US military servicemen reported a two- and four-fold increase in risk of UL musculoskeletal injury in people with traumatic unilateral and bilateral lower limb loss 1 year postamputation compared to a minor lower limb injury.^13^ Our study suggests that this increased risk of UL musculoskeletal injury results in increased odds for more UL disability 8 years post-lower limb amputation. It is important to note that people with lower limb loss in the ADVANCE cohort did receive UL-specific rehabilitation to mitigate future overuse musculoskeletal injuries and may have other important characteristics that effect their upper limb function, such as a nonamputation UL injury (see Suppl. Tab. A).

Wheelchair users rely on their ULs for weight-bearing and propulsion and commonly develop degenerative UL pathologies resulting in disability from about 12 years of wheelchair use.^14^ People with lower limb loss are likely to intermittently use a wheelchair complementary to their prostheses.^32^^,^^33^ We expect that the biomechanical demand on a wheelchair user’s ULs is higher than in a prosthesis user due to the additional demands of propulsion and performing daily overhead activities.^14^ The current increase in DASH score is small and not clinically significant but, as in wheelchair users, we expect that people with lower limb loss who use a prosthesis also deliver increased loads through their ULs and apply altered biomechanics through weight-bearing, transfer, and mobility activities that could affect their UL disability over time.^13–16^ Furthermore, we expect that people with bilateral lower limb loss will experience UL disability sooner and decline faster than people with unilateral lower limb loss due to more regular reliance on a wheelchair and more dependence on their ULs.

People with major and partial UL loss had significantly more UL disability than the Uninjured group and the Injured--Non-Amputee and Injured--Major Lower Limb Loss subgroups. The combined Inj-UL group had increased odds of having a higher DASH score more than 8 times greater than the Uninjured group, although the CIs were wide. Two recent studies on military personnel with UL loss with a similar follow-up time to this study both reported much higher mean DASH scores than this study, albeit in smaller populations.^3^^,^^4^ We expect participants in both other studies to have had access to similar levels of rehabilitation as the ADVANCE cohort, as both contain military personnel (except 2 civilians in 1 paper) injured in recent conflicts. The DASH questionnaire has been shown to be sensitive to rehabilitation interventions.^5^ Sabharwal et al included only people with transhumeral amputation being assessed for osseointegration, so higher scores may be expected as a result of high amputation level and presumed lack of tolerance of standard prosthetics.^3^ Pfister et al included 2 people with bilateral UL loss (both with a transradial and partial upper limb amputation), which could incur more difficulties.^4^ Our study included only people with unilateral UL loss and 5 participants with a transhumeral amputation whose DASH scores were generally higher than those with a transradial amputation, but not significantly so, and still much lower than elsewhere^3^ (see Suppl. Tab. B). Lower DASH scores could have been seen in our cohort due to handedness, though the dominant limb was more often amputated than the nondominant limb in our cohort. Other factors such as social support and concomitant injuries (eg, nerve damage, burns, traumatic brain injury) may also affect DASH score. These studies both report comparable DASH scores as seen in civilians with major UL loss across a similar period.^6^ Participants with UL loss in the ADVANCE cohort study have benefitted from high levels of rehabilitation and prosthetic services and report relatively low UL disability compared to similar military personnel and civilians with UL loss.

Contrary to reports that major UL amputation has a negative effect on mental health,^34^ adjunct mental health research on the ADVANCE cohort has shown a 118% increased relative risk for reporting a large amount of posttraumatic growth (positive psychological change following trauma) resulting directly from a major amputation (upper or lower limb) and reported similar mental health outcomes as the Uninjured group.^35^ The Major UL Loss group in this study contains 12 (75%) participants who also have bilateral lower limb amputations. Perhaps high levels of posttraumatic growth in this cohort contributes to better self-reported outcomes.

UL amputation increases the risk^11^ and prevalence^36^ of subsequent UL musculoskeletal injury, reduces shoulder and neck mobility^7^ and increases prevalence of neck and shoulder pain.^37^ This is due to altered biomechanics of the ipsilateral limb,^38^ compensatory movements of the contralateral limb and torso^38^^,^^39^ and potential for overreliance on the contralateral limb.^40^ This could result in an accelerated increase of disability long term for people with major and partial UL loss, compared to the remaining ADVANCE cohort groups.

Fewer studies report long-term outcomes for people with partial UL loss compared to major UL loss.^4^^,^^5^^,^^8–10^ A single military study included a subset of 2 people with partial UL loss with mean DASH scores of 45.2 at a mean of 6.5 years postinjury.^4^ Short-term outcomes have been reported in civilian populations reporting DASH scores between 7 and 47 up to 2 years after injury, depending on the amputation level.^5^^,^^8–10^ This study has demonstrated that people with partial and major UL loss have similar levels of UL disability, thus requiring similar quality and quantity of rehabilitation and access to advanced prosthetic technology regardless of perceived injury severity. Though numbers were small, participants with a partial hand amputation involving the thumb had the highest median DASH score of all people with UL loss (see Suppl. Tab. B). Lack of a thumb makes a pinch grip challenging, whereas major UL prosthesis users are likely to be able to achieve a pinch grip. Details of prosthesis use in people with partial UL loss was not captured, though anecdotal experience suggests uptake is low.

While not an original aim, important results were found for participants who sustained a combat injury requiring medical evacuation to the UK that did not result in limb loss. Adjusted regression analysis showed significantly increased odds (2.74 times) of having a higher DASH score than the Uninjured group. Basic categorization of this group’s UL injuries is included in Supplementary Table B, but further research is required to better understand their injuries to improve preventative screening, rehabilitation, and education to limit disability progression.

### Limitations

The main limitation of this study is the sole use of a patient-reported outcome measure and inclusion of people with comorbid lower limb loss in the Inj-ULmajor and Inj-ULpartial groups for statistical robustness means that potential influence of multiple limb loss on disability cannot be measured. The DASH questionnaire may not reflect technological advancements such as smartphones and speech-to-text innovations that are commonplace today and likely aid those with UL loss.

This young, highly rehabilitated military population with traumatic lower limb loss does not well reflect the general lower limb loss population, who may be older and have elective amputations for diabetic or vascular reasons.^41^ However, this population sustained widespread injuries beyond their limb loss status, which could incur more UL disability than the general lower limb loss population. Detail regarding musculoskeletal injuries sustained in the period between amputation and data collection that could have provided a more complete clinical picture.

### Conclusion

In conclusion, there is some evidence for more UL disability in people with lower limb loss compared to an Uninjured comparison group 8 years after injury, but it is not currently clinically significant. People with major and partial UL loss have more UL disability than other Injured subgroups and the Uninjured control group, but this is low compared to other reported populations, perhaps due to high levels of prosthesis use, intense rehabilitation, and good mental health. The ADVANCE study will continue to follow this population for the next 20 years to monitor how UL disability changes over time, which could impact rehabilitation of people with lower and UL loss.

## Supplementary Material

2023-0799_R1_Suppl_Material_pzae082

## Data Availability

Data are available upon reasonable request. Given the sensitive nature of the participants, the data have not been made widely available. Requests for data will be considered on a case-by-case basis and subject to UK Ministry of Defence clearance. The views expressed are those of the authors and not necessarily those of the NHS, the NIHR, or the Department of Health.
