# Supplementary material for: Upper Limb Function in People With Upper and Lower Limb Loss 8 Years Postinjury: The Armed Services Trauma Outcome Study (ADVANCE) Cohort Study
Source: Phys Ther. 2024 Jul 2;104(10):pzae082. doi: 10.1093/ptj/pzae082 (PMC11491512; doi:10.1093/ptj/pzae082)
Supplement: 2023-0799_R1_Suppl_Material_pzae082 [file 2023-0799_r1_suppl_material_pzae082.pdf]

Supplementary Files

Table A

Disability of the Arm, Shoulder, and Hand (DASH) questionnaire scores for the Injured – Non-Amputee (Inj-NA) and Injured – Major Lower Limb Amputee (Inj-LL) groups categorized by their worst upper limb injury. Non-amputee upper limb injuries were categorized using the UK Joint Theatre Trauma Registry, and participants were grouped according to their worst upper limb injury. The Abbreviated Injury Score (AIS) score was used to group soft tissue injuries into mild (AIS ≤1) and moderate/severe (AIS >1).

	**DASH n(%) median (range)**	
	**Inj-NA***	**Inj-LL**
No upper limb injury	200 (49.50)<br>0.00 (0.00-72.41)	43 (39.45)<br>0.00 (0.00-31.67)
Mild soft tissue wound (AIS≤1)	64 (15.84)<br>3.75 (0.00-58.33)	23 (21.10)<br>0.83 (0.00-55.83)
Moderate/Severe soft tissue wound (AIS>1)	22 (5.45)<br>7.92 (0.00-59.17)	8 (7.34)<br>3.34 (0.00-8.33)
Nerve injury	20 (4.95)<br>17.50 (3.33-51.67)	6 (5.50)<br>4.59 (0.00-29.17)
Fracture/dislocation	57 (14.11)<br>14.17 (0.00-67.50)	29 (26.61)<br>3.33 (0.00-26.67)

*Sufficiently detailed injury data missing for 42 Inj-NA group participants.
AIS = Abbreviated Injury Score

Table B

Disability of the Arm, Shoulder, and Hand (DASH) questionnaire scores for Injured – Partial Upper Limb Amputees (Inj-ULpartial) and Injured – Major Upper Limb Amputee (Inj-ULmajor) by level of amputation.

**Level of upper limb amputation**	**DASH**<br>**n**<br>**median(range)**
Transphalangeal II-V	34<br>11.87 (0.00-86.67)
Transphalangeal I +/- Transphalangeal II-V	8<br>26.67 (0.00-56.67)
Transradial	11<br>7.41 (0.00-45.69)
Transhumeral	4<br>25.00 (12.50-44.17)
Shoulder disarticulation	1<br>15.83 (n/a)
